# Extraction Optimization, Characterization and Biological Activities of Polysaccharide Extracts from *Nymphaea hybrid*

**DOI:** 10.3390/ijms24108974

**Published:** 2023-05-18

**Authors:** Hui-Min Liu, Wei Tang, Sheng-Nan Lei, Yun Zhang, Ming-Yan Cheng, Qing-Lei Liu, Wei Wang

**Affiliations:** 1 School of Perfume & Aroma and Cosmetics, Shanghai Institute of Technology, Shanghai 201418, China; szliuhm@sit.edu.cn (H.-M.L.); toei.tang@outlook.com (W.T.);; 2 Engineering Research Center of Perfume & Aroma and Cosmetics, Ministry of Education, Shanghai 201418, China

**Keywords:** *Nymphaea hybrid*, extraction, antioxidant, bioactivities

## Abstract

In this study, polysaccharide–rich *Nymphaea hybrid* extracts (NHE) were obtained using the ultrasound-assisted cellulase extraction (UCE) method optimized by response surface methodology (RSM). The structural properties and thermal stability of NHE were characterized by Fourier-transform infrared (FT–IR), high–performance liquid chromatography (HPLC) and thermogravimetry–derivative thermogravimetry (TG–DTG) analysis, respectively. Moreover, the bioactivities of NHE, including the antioxidant, anti–inflammatory, whitening and scratch healing activities were evaluated by different in vitro assays. NHE conveyed a good ability to scavenge against the 2,2-diphenyl-1-picrylhydrazyl (DPPH) free radicals and inhibit the hyaluronidase activity. NHE can effectively protect the HaCaT cells against oxidative damage by inhibiting the intracellular reactive oxygen species (ROS) production in the H_2_O_2_ stimulation assays and promoting the proliferation and migration in the scratch assays. In addition, NHE was proven to inhibit melanin production in B16 cells. Collectively, the above results seem to be the evidence needed to promote the potential of NHE to be regarded as a new functional raw material in the cosmetics or food industries.

## 1. Introduction

*Nymphaea hybrid* (NH), a water lily that belongs to the Nymphaeaceae family, is known as the “Nine Ranks Perfume Lotus” for its nine colors after being cultivated in China in the 1970s [1,2,3]. The reason why its flowers have many colors is that the petals precisely contain a lot of anthocyanins and flavonoids [3]. In addition, NH flowers also have polysaccharide crude fiber, protein and other components. These ingredients make NH have more practical applications. In the theory of dermatology, ultraviolet (UV) stimulation, inflammatory stimulation, viruses and bacteria can increase the level of ROS free radicals, create oxidative stress and even cause injuries to skin cells, such as cell aging, necrosis and apoptosis. In recent studies, the alcohol extracts (mainly flavonoids and polyphenols) from NH have been proven to have antioxidant activity, including strong scavenging activities on free radical and lipid peroxidation inhibition ability, indicating to some extent that the alcohol extract from NH can act as anti–aging active material in the field of cosmetics [4,5]. Moreover, various extracts of NH are also used in various fields such as food [3,6] and medicine [4,7]. However, most of the research on NH has been focused on alcohol extracts, which often face solubility, stability and other problems for application in the aqueous systems. Polysaccharides are found in almost every plant and play crucial biological activities in the organism, such as immune regulation, regulation of cell growth and reproduction, antioxidation and anti–aging functions [8]. Polysaccharides are rich in many parts, such as the flower, stem and leaf of the *Nymphaea hybrid*. However, the polysaccharide extracts from the *Nymphaea hybrid* have not been systematically studied in their composition and bioactivities, particularly on a cellular level.

As a distinctive biomolecule found in nature, polysaccharides have aroused the interest of numerous researchers owing to their potential applications. Some extracts derived from traditional Chinese medicinal plants, which contain polysaccharides, have been explored and utilized as anticancer agents [9]. At present, decoction [10], ultrasonic [11], enzymolysis [12], flash extraction [13], supercritical fluid extraction method [14], etc. are relatively efficient extraction methods for polysaccharides. Remarkably, the ultrasonic method and enzymatic hydrolysis method have the advantages of convenient operation and low cost on the basis of a good extraction rate. The enzymatic method can destroy the cell wall to make it easier for the solvent to penetrate cells [15], and the extraction effect may be enhanced further by the mechanical fluctuation and cavitation effect of ultrasonic treatment [16]. Hence, the combination of both can often achieve better extraction results.

The response surface method is an efficient method for designing and evaluating experiments to optimize extraction conditions, exploring multiple factors and their interactions, which simplifies the number of trials and optimization processes [17]. It is a good way to explore high yield under optimal conditions.

The aims of this study are (1) to obtain the optimal extraction conditions of NHE by employing the UCE coupled with RSM; (2) to characterize the structure of NHE by FT–IR and HPLC and determine the thermal properties using TG–DTG analysis; (3) to measure the various biological activities of NHE using in vitro chemical and cellular methods, which provide the experimental basis for the follow-up research, production and application of NHE.

## 2. Results and Discussion

### 2.1. Single Factors Experiment Analysis

To explore the influence of different variables, we developed an experimental program with four factors and five levels in the single factors experiments to determine the influence on yields of total sugar. The optimal conditions based on the single factor assays were determined, namely, an ultrasound time of 30 min, a liquid-to-solid ratio of 1:30 mL/g, an enzymatic temperature of 50 °C and an enzyme content of 3% of the substrate.

**Figure 1:**
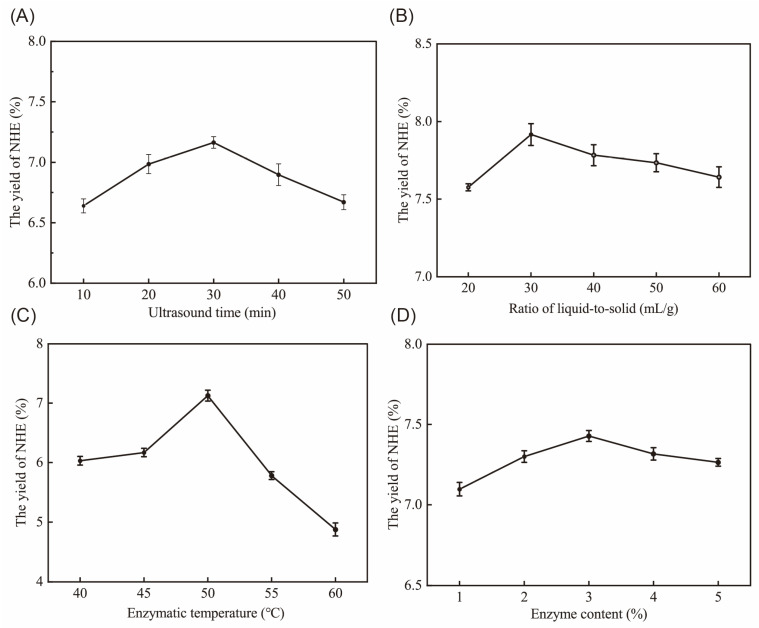
The effect of different ultrasound times (**A**), ratio of liquid-to-solid (**B**), enzymatic temperature (**C**) and enzyme content (**D**) on the yield of NHE.

In Figure 1A, the optimal yield of NHE was obtained at an ultrasound time of 30 min. There was an increase in NHE yield with increasing ultrasound time, as NHE dissolution increased. However, the yield decreased when the time exceeded 30 min due to the assimilation of cavitation energy and an increase in impurities. The liquid-to-solid ratio assay, as depicted in Figure 1B, revealed similar trends. An excessive solvent could assimilate cavitation energy from the extraction process, leading to a reduction of the concentration of cellulase and substrate [18]. Moreover, Figure 1C,D demonstrated that enzymatic hydrolysis with cellulase, which destroys the cell wall, enhanced the yield of NHE [19]. Temperature is a key factor for enzymatic hydrolysis, which might influence the activity of cellulase and the solubility of polysaccharides. Hence, the yield of NHE exhibits a propensity to decline when the hydrolysis temperature surpasses 50 °C, relying on the optimal conditions of cellulase. Generally, the enzymes exhibited the highest activity under specific temperature and pH conditions, but a further increase in enzyme concentration did not result in a significant improvement, and then it started to decline. This is in line with the previous trend of enzymatic extraction [20].

### 2.2. Optimization of Extraction Parameters

#### 2.2.1. Optimization of NHE Yield by RSM

According to the results of these batch experiments, three factors were further selected as the independent variables to optimize the extraction process by BBD and their effects on the yield of total sugar, and the interactions were evaluated. The three independent variables were ultrasound time (X_1_, A), liquid-to-solid ratio (X_2_, B) and the enzymatic hydrolysis temperature (X_3_, C). The results are shown and analyzed in Table 1.

The predicted response variables (Yield, Y) could be demonstrated by the equation as follows:

(1) $$Y=-39.39+0.28A+0.53B+1.47C+1.21\times 10^{-3}\mathrm{AB}+4.16\times 10^{-3}\mathrm{AC}\;-3.82\times 10^{-3}\mathrm{BC}-8.37\times 10^{-3}A^{2}-5.99\times 10^{-3}B^{2}-0.02C^{2}$$

Analysis of variance (ANOVA) was used to calculate the different factors affecting the yield, and a subsequent F–test was conducted to determine the statistical significance of the regression [21]. The *p*-values of each factor were used to assess their contribution and significance [22]. Table 2 indicates that the model had a significant effect on the yield, as evidenced by the low *p*-value (<0.0001) and substantial F-value (34.47). The *p*-value of the Lack of Fit was >0.05, with a value of 0.2558. The lack of fit is an indication of the failure of a model representing the experimental data in which points are not included in the regression or variations in the model, and random error cannot be accounted for [20,23]. Therefore, the results showed that the regression model was statistically significant in fitting the experimental data, and the model was meaningful [24]. The *p*-value (*p* < 0.05) of linear coefficients of A, B and C, cross-product coefficients of AC, BC and quadratic coefficients of A^2^, B^2^ and C^2^, suggested that the model developed for the response was significant [25]. In addition, the correlation coefficient value (R^2^ = 0.9779) showed that more than 97.79% of the response variabilities were explained by the model. The adequate precision was 16.60, which indicated that the model was an adequate signal [26]. To sum up, these results suggested that the influence of three factors on the yield of total sugar in extraction was an interacting relationship rather than a simple linear relationship.

#### 2.2.2. Analysis of Response Surfaces

**Figure 2:**
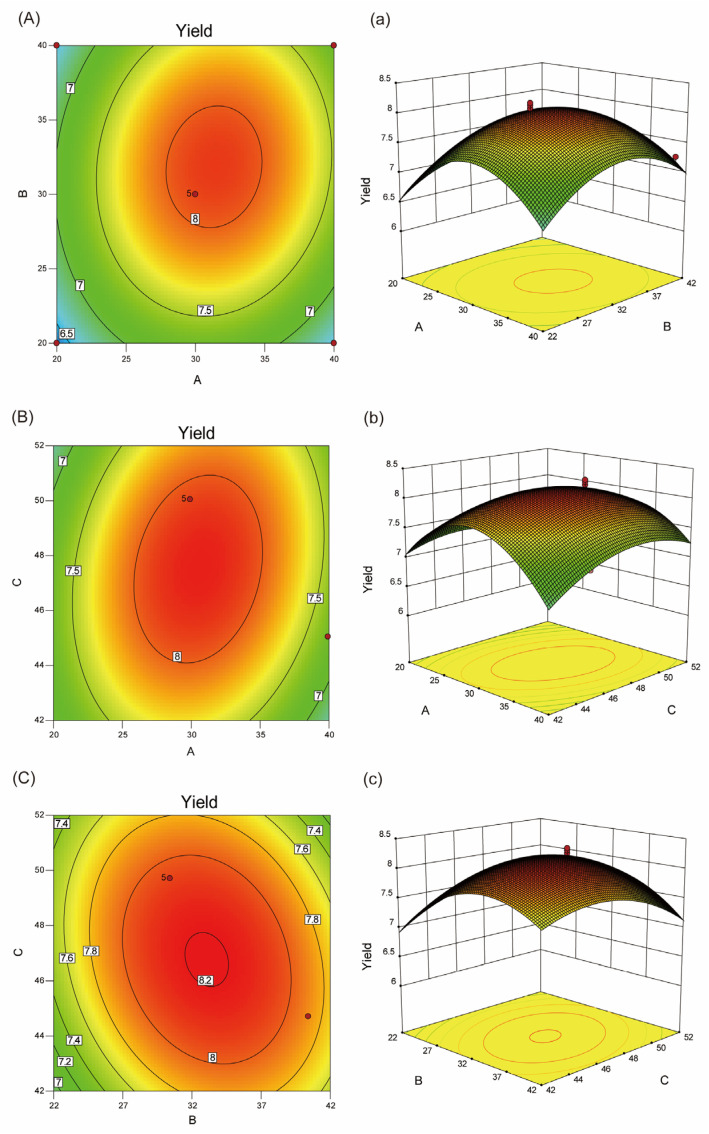
Response surface plots and contour plots showed the interactions between different extraction parameters. (**A**) 2D contour plot of ultrasound time and liquid-to-solid ratio. (**a**) 3D response surface plot of ultrasound time and liquid-to-solid ratio. (**B**) 2D contour plot of ultrasound time and enzymatic hydrolysis temperature. (**b**) 3D response surface plot of ultrasound time and enzymatic hydrolysis temperature. (**C**) 2D contour plot of liquid-to-solid ratio and enzymatic hydrolysis temperature. (**c**) 3D response surface plot of liquid-to-solid ratio and enzymatic hydrolysis temperature. The red dots represent the actual values of yield under specific conditions.

Design-Expert 10.0.7 software was used to plot the three-dimensional response surface diagram (3D Surface) that showed the interactions and experimental levels of three parameters on the yield of total sugar [8]. Based on the results, the shapes of the plots changed with the different impacts of the different variables in the images. Elliptical plots showed significant influences on the relevant parameters [27]. The best horizontal range was the top of the elliptical or its vicinity. The steeper curve of the 3D surface indicated a greater effect on the experimental results [28]. According to these 3D surfaces, the interaction of ultrasound time and liquid-to-solid ratio (Figure 2A,a), the interaction of ultrasound time and enzymatic hydrolysis temperature (Figure 2B,b), the interaction of enzymatic hydrolysis temperature and liquid-to-solid ratio (Figure 2C,c) together formed elliptical contours, indicating perfect interaction between the independent variables [20,29]. The *p*-values for variables AB, AC and BC were 0.0176, 0.0360 and 0.0492, respectively, all falling below the significant threshold of 0.05. This suggested a significant interaction between any two factors. To explore the order of interactions and their effects on the response value, F-values and contour maps were observed, leading to the conclusion that the order of interactions in terms of the greatest effect on the response value was BC > AC > AB. These findings implied that hydrolysis temperature (C) plays a major role in the yield of NHE in this extraction process. This also highlighted the importance of enzymatic hydrolysis in ultrasound extraction, as it directly interacts with the liquid–solid ratio and extraction time.

Based on the BBD experiments and the second-degree polynomial equation, the optimal process conditions were obtained as follows: the ultrasound time was 30.69 min, the liquid-to-solid ratio was 32.69 mL/g and the enzymatic hydrolysis temperature was 47.15 °C. The predicted yield of total sugar was 8.22 ± 0.08% (*w*/*w*) under optimal conditions. On this basis, the yield of dry purified NHE reached 5.26 ± 0.13% (*w*/*w*). The extraction rate of NHE was not systematically studied, but it is significantly higher than the polysaccharide yield of carpel of Nymphaea odorata (3.19%) [30].

#### 2.2.3. Verification

In order to verify the optimum combination of the extraction conditions, five parallel trials were carried out under the optimal conditions (A = 30.69 min, B = 32.69 mL/g, C = 47.15 °C). The yield of total sugar was 8.11 ± 0.08%, quite close to the predicted value (8.22 ± 0.16%), and it proved the validity of the model.

### 2.3. Component Analyses

The obtained NHE powder was analyzed by the below-mentioned Section 3.4, and the results are shown in Table 3. It can be seen that NHE is rich in a large number of sugars, basically removing proteins, polyphenols and other substances. The neutral sugars and uronic acids obtained by hydrolysis reach 82.99% of the dry weight. From that, it can be speculated that the powder is a polysaccharide-rich water extract, and the structure and biological activity were analyzed and determined.

**Figure 3:**
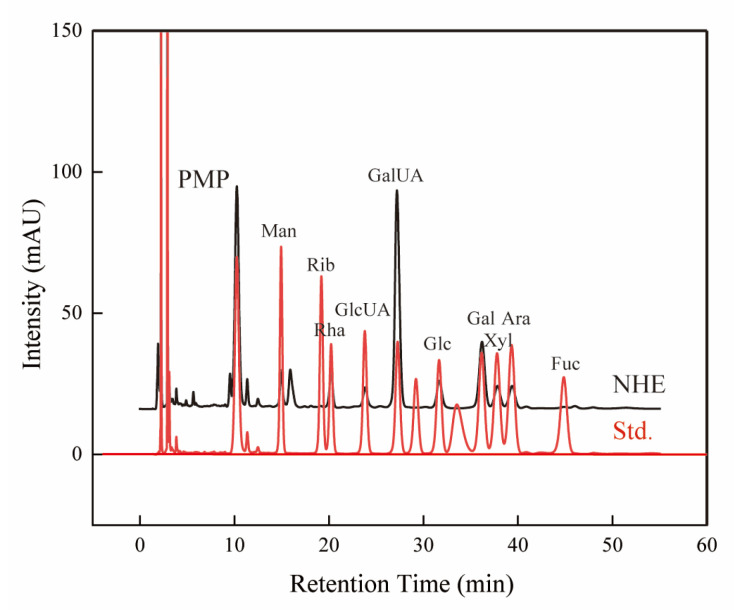
HPLC Chromatograms of NHE (black line) and standard monosaccharide standard (red line).

It is widely known that the monosaccharide composition is a significant factor closely related to the bioactivities of natural polysaccharides [31]. The monosaccharide composition of crude polysaccharides was identified by HPLC with PMP derivatives [32,33], and their existence and content were determined by comparing several standards as shown in Figure 3 and Table 4. The results showed that GalUA and Gal were the two major monosaccharides of NHE, with the highest content being 47.31% and 18.61%, respectively. In addition, other eight monosaccharides with lower content, i.e., Man, Rib, Rha, GlcUA, Glc, Xyl, Ara and Fuc, were identified and quantified. Similarly, these monosaccharides were also found as the composition of other natural polysaccharides [33,34,35]. It is worth noting that different monosaccharide compositions are relevant to the difference in bioactivities [36,37].

### 2.4. Fourier Transform Infrared Spectroscopy Analysis

**Figure 4:**
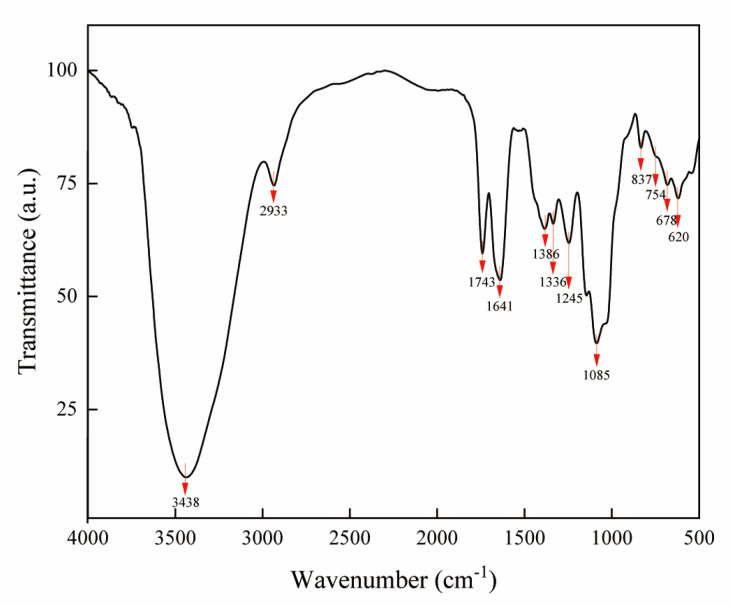
FT–IR spectrum of NHE. The red arrows represent wavelength markers that may have absorption peaks for characteristic organic groups.

FT–IR spectroscopy is a very practical means for detecting characteristic organic groups in complex components [38]. As illustrated in Figure 4, the FT–IR spectrum of NHE displayed the particular signals of polysaccharides. The presence of hydroxyl groups was proven by the broad and intense peaks at 3438 and 1641 cm^−1^ assigned to the stretching vibration of O–H due to intermolecular or intramolecular hydrogen bonds [39] and the peak at 1336 cm^−1^ attributed to the in-plane flexural vibration of O–H [40]. The strong absorption peak at 1085 cm^−1^ was caused by flexural vibrations of O–H and C–O [41]. The sharp absorption peak at 1743 cm^−1^ could be the symmetric stretching vibration of C=O which was common and present in carbohydrates. Especially, the peak at around 3200~3600 cm^−1^ was attributed to O–H stretching of the –COOH group on the galacturonic acid backbone [42], which corresponded with the above results of HPLC. The weak absorption peak at 2933 cm^−1^ belonged to the C–H and C–H_2_ stretching vibration signal of polysaccharide methylene [27,37] and the absorption peak near 1386 cm^−1^ certified again the presence of C–H. The peaks at 837, 754 cm^−1^ (905~675 cm^−1^) indicated the presence of C–H of the aromatic ring [43,44]. NHE showed a new strong absorption at 1245 cm^−1^, which was caused by the sulfate group [45]. These characteristic organic groups are the important structural basis for NHE to exert its biological activity.

### 2.5. Thermal Property Analysis

**Figure 5:**
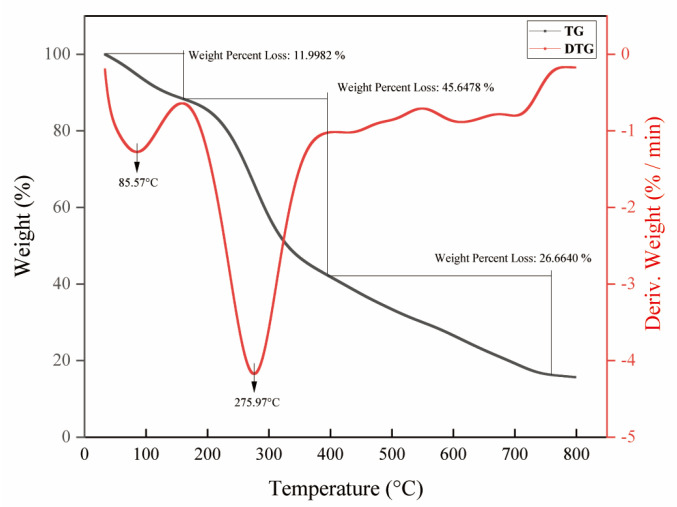
TG–DTG curves of NHE. The black arrow represented the temperature of the fastest weightlessness in the stages.

The thermal stability of biomolecules can be employed to measure quantitatively the change in material mass with time and temperature during dehydration, decomposition and oxidation [37,46,47]. The thermodynamic property of polysaccharides is also a fundamental property of industrial biomolecular applications [37]. In this experiment, the thermal properties were determined by the combination of TG–DTG analysis. The pyrolytic curves of NHE were divided into three main steps of weight loss as shown in Figure 5. The first stage was in the temperature range of 33.2~163.6 °C, during which the weight loss rate reached 12.0%. The result might be attributed to the evaporation of the free water and bounding water to the polysaccharides [48,49,50]. The second stage occurred in the temperature range of 163.6~395.3 °C, during which the weight loss was the most serious. When the temperature rose to 275.97 °C, the weight of polysaccharides began to decrease sharply, which might be caused by the depolymerization and decomposition of polysaccharides [51]. In addition, there was some weight loss in the third stage of 395.3~759.8 °C, which might be due to the decomposition of macromolecular or inorganic impurities [49,52]. Consequently, these results showed that NHE had relative thermal stability below 275.0 °C [37,53]. The bioactivity of NHE was not influenced by temperature during extraction and purification. Further, the results provide a foundation for future research into NHE applications, including gel, food and emulsion preparation.

### 2.6. DPPH Radical Scavenging Activity

**Figure 6:**
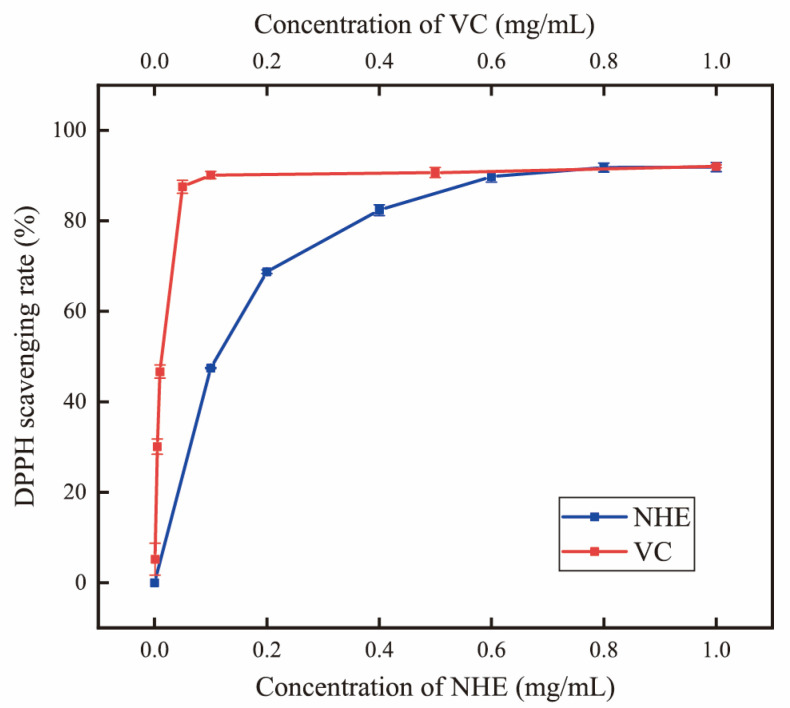
Determination of DPPH radical scavenging rate (%).

The DPPH scavenging assay is a widely used method for evaluating the antioxidant capacity of biomolecules [54]. As shown in Figure 6, the DPPH radical scavenging rate increased from 27.39% to 91.90% when the NHE concentration increased from 0.1 to 1.0 mg/mL. The DPPH radical scavenging rate of NHE was significantly increased with the concentration, indicating that the NHE had certain antioxidant activity while the effect was weaker than VC. The IC_50_ of the NHE and VC for DPPH radical scavenging activity were 0.0817 and 0.008 mg/mL, respectively. Based on the results, NHE exhibited significantly higher antioxidant activity than many published polysaccharides [37,55,56,57]. The scavenging effect of DPPH free radicals can, to some extent, indicate the sample’s antioxidative or anti-aging activity in cells or the body [58]. The results suggested that NHE could be considered a potential natural antioxidant, making it an essential component in the fields of food and cosmetics industries.

### 2.7. Hyaluronidase Activity Inhibition 

**Figure 7:**
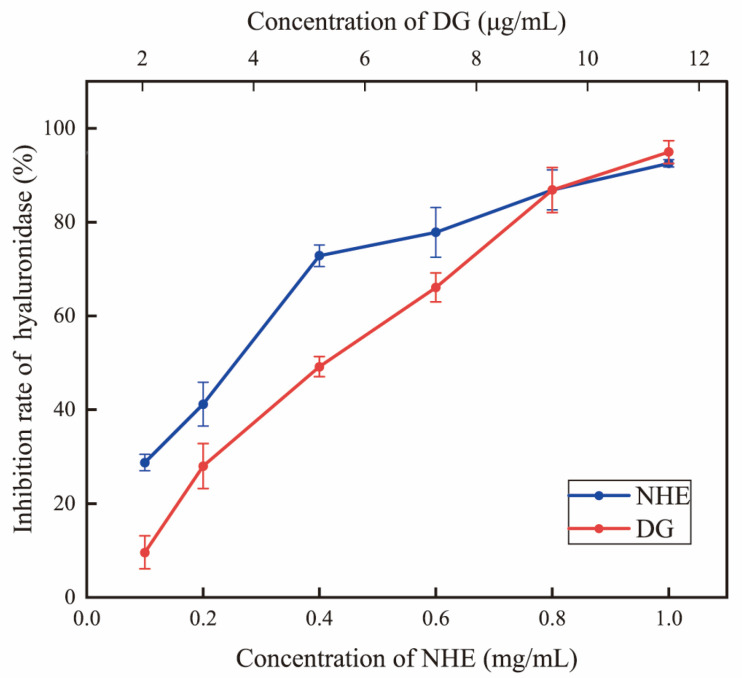
Determination of hyaluronic acid scavenging rate (%).

Hyaluronidase activity inhibition is usually used to assess the anti-inflammatory and anti-allergenic effects of active substances [59]. Hyaluronic acid plays a significant role in tissue regeneration, including skin healing and wound repair, inflammation response and angiogenesis [60]. In this study, the hyaluronidase inhibition rate increased obviously with the increase in the sample concentration indicating that the hyaluronidase inhibition ability was concentration-dependent (Figure 7). The IC_50_ value of NHE in the hyaluronidase inhibition assays was 0.2557 mg/mL, while in the control group, the hyaluronic acid scavenging rate of 10 μg/mL Dg was 94.23 ± 1.72%, indicating that NHE displayed some anti-inflammatory ability but it is weaker than that of Dg. This biological activity is similar to that reported in previous studies, which mainly contain polysaccharides [61,62,63]. This might be related to some monosaccharides, which played an inhibitory role in enzyme activity [62]. Similar studies have shown this biological activity in extracts containing polysaccharides in rose petals [63]. In a word, NHE could be regarded as a potential ingredient in applications that promote wound healing. This provided a way to develop natural active products in the future.

### 2.8. Effect of NHE on ROS Level of HaCaT Cells

**Figure 8:**
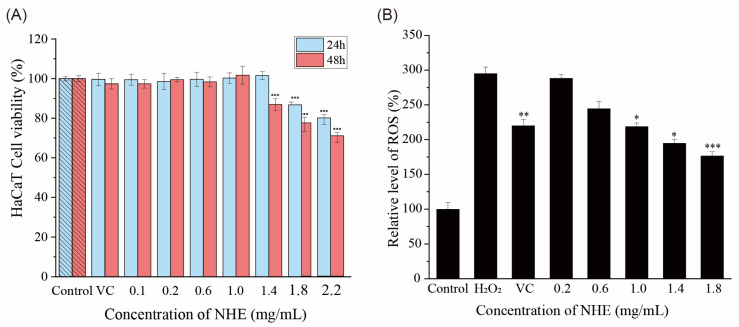
(**A**) The HaCaT cell viability with different NHE concentrations for 24 h and 48 h. (**B**) Average intensity of fluorescence in HaCaT cells. *p* < 0.05, *p* < 0.01 and *p* < 0.001 were performed *, ** and ***, respectively.

The cell viability was monitored using CCK-8 colorimetric to determine the cytotoxicity of NHE to HaCaT. As for HaCaT cells in Figure 8A, when the cells were cultured in the NHE concentration range of 0.1~1.0 mg/mL, there was no cytotoxicity for the exposure time of 24 h or 48 h with stable cell viability. The cell viability decreased when exposed to NHE with a higher concentration (1.4~2.2 mg/mL). The cytotoxicity increased with the increase in the exposure time. Therefore, the HaCaT cells were selected to culture with NHE in the concentration range of 0.2~1.8 mg/mL for 24 h, and under this condition, the cell viability was higher than 80% of the control group. The safety and efficacy of NHE were further demonstrated via cell activity experiments, hence providing a theoretical foundation for its future practical application in industries.

H_2_O_2_ was used as an inducer of the production of intracellular ROS at certain concentrations. The cytotoxicity of H_2_O_2_ on HaCaT cells was verified by CCK-8 assays on the premise of maintaining certain cell viability. According to previous experiments [5], 100 μM H_2_O_2_ was selected to stimulate HaCaT cells for 2 h in this cellular injury model.

**Figure 9:**
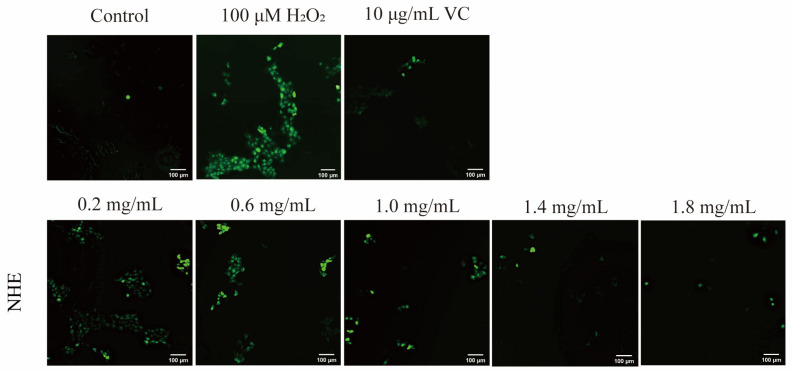
Distribution of ROS in HaCaT cells. Intracellular ROS levels and distribution were measured with fluorescence imaging using the DCFH–DA probe in cells cultured in the presence of NHE (0.2, 0.6, 1.0, 1.4 and 1.8 mg/mL) for 24 h. Every sample was stimulated with 100 μM H_2_O_2_ for 2 h and set as a blank control group and a positive group using Vitamin C (10 μg/mL). The scale bars were 100 µm.

The intracellular ROS level, as indicated by the fluorescence intensity of DCF, was measured with an enzyme-labeling or fluorescence microscope. As shown in Figure 9, HaCaT cells were cultured first with 0.2~1.8 mg/mL NHE and then treated with 100 μM H_2_O_2_ for 2 h. Compared with the negative control group (100 μM H_2_O_2_ treatment only), the fluorescence intensity of DCF in the NHE treatment groups obviously decreased, indicating that NHE pretreatment in the concentration range can efficiently inhibit the adverse effect. The inhibiting effect of NHE on ROS production was enhanced in a dose-dependent manner. Additionally, the effect was similar to the positive control group of VC (10 μg/mL). In Figure 8B, the ROS intensity of the VC pre-treatment group was 220.17 ± 9.12% (*p* < 0.01) and those of groups pre-treated with the different concentrations (0.2~1.8 mg/mL) of NHE were 288.35 ± 5.25% (*p* > 0.05), 244.52 ± 10.56% (*p* > 0.05), 218.78 ± 5.22% (*p* < 0.05), 194.78 ± 5.52% (*p* < 0.05), 176.70 ± 5.75% (*p* < 0.001), respectively, while the ROS intensity of the group only treated with the H_2_O_2_ reached 295.17 ± 9.29%. In summary, NHE can effectively inhibit ROS production and contribute to the protection of HaCaT cells against the oxidative damage of H_2_O_2_.

### 2.9. Effect of NHE on Melanin Production of B16 Cells

**Figure 10:**
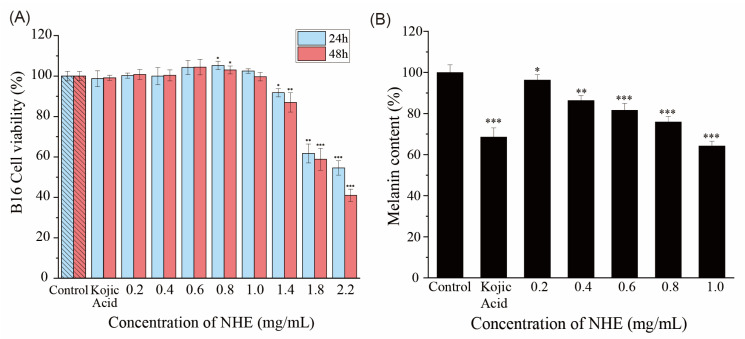
(**A**) Effects of NHE on B16 cell viability. (**B**) Melanin content with NHE concentration. The *p* < 0.05, *p* < 0.01 and *p* < 0.001 were performed *, ** and ***, respectively.

Melanin plays a crucial role in skin pigmentation, which is one of the main attributes of skin aging. Hence, one of the ways to mitigate skin pigmentation is to inhibit melanin synthesis [64]. It is widely recognized that an effective way of whitening is to inhibit the activity of tyrosinase and the production of melanin [65]. As shown in Figure 10A, the exposure to NHE in a relatively low concentration (0.1~1.0 mg/mL) for 24 h or 48 h did not produce an adverse influence on cell viability, which was proven by the stable cell viability. However, with the increase in NHE concentration and the extension of culture time, the cytotoxicity of cells increased, causing cell shedding and even death. Therefore, compared with the blank control group, the presence of NHE inhibited the production of melanin and the melanin content decreased with the increase in the NHE concentration in Figure 10B. When the NHE concentration was 0.2, 0.4, 0.6, 0.8 and 1.0 mg/mL, the melanin relative content of B16 cells was 96.32 ± 2.58%, 86.33 ± 2.34%, 81.63 ± 3.33%, 75.92 ± 2.75%, 64.29 ± 2.25%, respectively. Kojic acid is a well-known whitening ingredient and the melanin content in the positive (20 μg/mL kojic acid) group was 68.57 ± 4.47%, which was close to the melanin content in the presence of 1.0 mg/mL NHE. These results indicated that NHE had a certain whitening effect in higher concentrations. This may be due to the fact that NHE can inhibit melanin synthesis and tyrosinase activity [66,67]. This kind of function is relatively rare in previous polysaccharide research. In contrast, active substances with flavonoids or polyphenols are more commonly found [68,69,70]. This characteristic reflects the advantage of NHE in skin whitening, coupled with its water-solubility feature, making it a valuable product for applications in the cosmetic and food industries.

### 2.10. Re-Epithelialization of NHE by Scratch Assay

**Figure 11:**
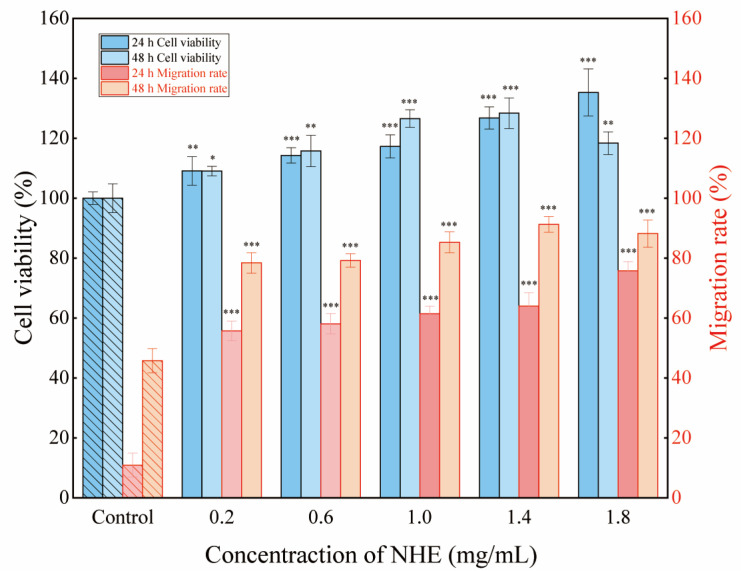
The cell viability and migration rate with concentrations of NHE. The right columns (red border) showed the migration of rate at 24 h and 48 h after treatment with different concentrations of NHE. Response of untreated cells (Control) was considered as 100%. The cells were cultured in DMEM medium with free FBS in the assay. The *p* < 0.05, *p* < 0.01 and *p* < 0.001 were performed *, ** and ***, respectively.

**Figure 12:**
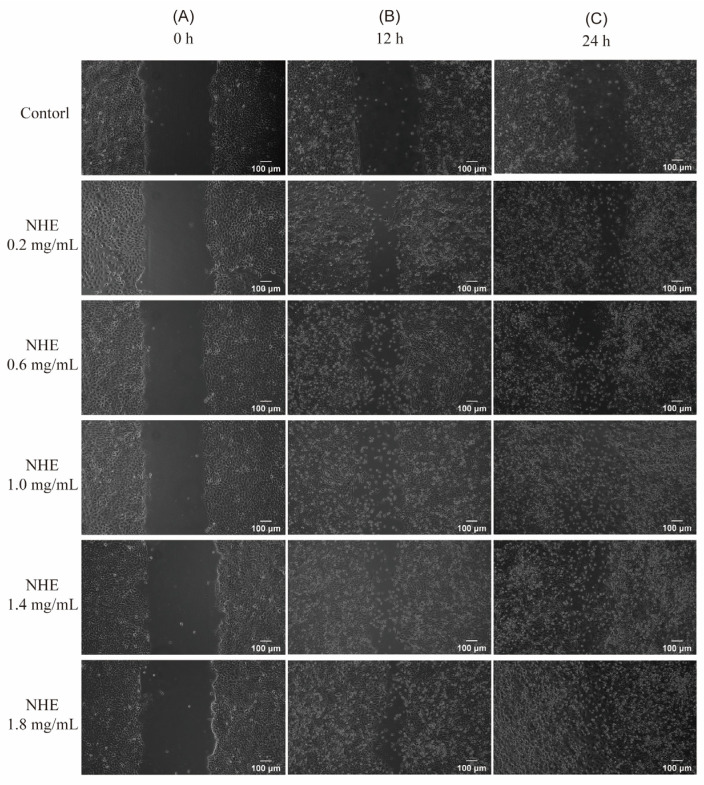
Effects of NHE on scratch wound healing of HaCaT cells at different times. Photos were taken at 0 h (**A**), 24 h (**B**) and 48 h (**C**) after treatment with NHE. Scale bars were equivalent to 100 µm.

The rate of cell migration is a crucial determinant in skin wound healing. Hence, investigating factors that influence epidermal keratinocyte and dermal fibroblast migration may help develop targeted therapies to improve cutaneous wound healing [71]. Figure 11 presents the percentage of cell viability and migration rate for NHE as a ratio to the untreated control [72]. The microscopic images depicting the wound gap closure after 0, 24 and 48 h treatment with NHE are shown in Figure 12A–C, respectively.

Consequently, these results can verify whether NHE could promote the rate of wound healing in HaCaT cells via scratch assays. In the presence of 0.2~1.4 mg/mL NHE, cell proliferation and migration enhanced obviously with the increase in NHE concentrations after the cells were cultured for 24 h or 48 h compared with the blank control group. The migration rates almost exceed 80% at multiple NHE concentrations and reached a maximum of 91.25% at NHE concentrations of 1.4 mg/mL. It was evident that the presence of NHE led to a notable improvement in wound gap healing in contrast to the untreated control of HaCaT cells. Therefore, it was proved that NHE is safe and effective in promoting wound healing and tissue regeneration [72,73,74]. This is also consistent with the above results demonstrating the inhibitory effects on hyaluronidase activity. These findings suggest that NHE has certain effects on promoting wound repair and anti-inflammation.

In addition, according to the above experimental results, it is reasonable to postulate that NHE has great potential as an anti-aging substance. The good antioxidant activity, inhibition of melanin production and promotion of epidermal cell migration are crucial features of anti-aging [58]. Although the specific anti-aging pathways for NHE are not yet clear, further research in this direction may be immensely valuable.

## 3. Materials and Methods

### 3.1. Materials and Reagent

*Nymphaea hybrid* flowers were supplied by a local supplier (Liandao Agricultural Co. Ltd., Shanghai, China). The fresh flower petals were washed with ultrapure water to remove any adhering soil or leaves, freeze-dried and then crushed by a shredder and sifted through 50 mesh. After degreasing with petroleum ether, the insoluble powder was dried in an oven at 50 °C and stored at a low temperature until the extraction procedure.

Methanol, formic acid and acetonitrile were of chromatographic grade. The standards were purchased from Sigma (St. Louis, MO, USA), besides Mannose (Man), ribose (Rib), rhamnose (Rha), glucuronic acid (GluUA), galacturonic acid (GalUA), N-acetyl-glucosamine (N-Glu), glucose (Glu), N-acetyl-galactosamine (N-Gal), galactose (Gal), xylose (Xyl), arabinose (Ara) and fucose (Fuc). The HaCaT cells and B16 mouse melanoma cell lines were purchased from the Shanghai Cell Bank of Chinese Academic of Science (Shanghai, China).

### 3.2. Preparation of NHE and Single Factors Experiment

According to the pre-experimental screening of cellulase (10,000 U/g, Macklin, Hampshire, UK), pectinase (30,000 U/g, Macklin) and papain (200 U/mg, Macklin), we finally selected cellulase to assist in increasing yield due to the highest sugar content. Cellulase significantly damages the cellulose of plant cells, allowing bioactive substances to flow out and increasing the yield. The UCE method was used for the preparation of NHE using the powder of *Nymphaea hybrid* flower as starting material. Batch preliminary experiments with different extraction conditions are as follows: the degreased powder (1.0 g) was extracted by solvents in ultrasound time (10~50 min) after enzymatic hydrolysis. The conditions for enzymatic hydrolysis are an enzymatic temperature of 40~60 °C, enzyme content of 1~5% and a liquid-to-solid ratio of 20~60 mL/g. The yield of total sugar in the extraction was detected using the phenol-sulfuric acid method, which was used as an indicator for NHE acquisition. Most of the extraction solutions were concentrated by reducing pressure. Four times the volumes of absolute alcohol were added for precipitation of crude polysaccharide at 4 °C for 12 h. The precipitate was collected by centrifugation and lyophilization to obtain the crude polysaccharide. The lyophilized powder was reconstituted by adding a small amount of water, then the protein was precipitated under trichloroacetic acid (TCA). Finally, the supernatant was concentrated by reducing pressure and vacuum freeze-drying to obtain NHE.

### 3.3. Response Surface Experiment

The Box–Behnken design (BBD) is an efficient design for a response surface methodology in three-level full factorial designs [75]. Based on the results of batch experiments, three variables and three levels were selected, and the BBD was applied to statistically optimize the extraction conditions. The three independent variables were considered as follows: ultrasound time (X_1_, A), liquid-to-solid ratio (X_2_, B), enzymatic hydrolysis temperature (X_3_, C). The response variable was the yield of total sugar represented the concentration of NHE as the result. The yield was determined as follows:

$$Yield\;\left(\%\right)=\frac{C\times V}{W}\times 100\%$$

where, *C* is the total sugar concentration (mg/mL); *V* is the extraction volume (mL); *W* is the weight of dried *Nymphaea* Hybrid powder (mg).

The crude extracts of NH obtained under optimal conditions were further purified and dried to acquire the NHE.

### 3.4. Determination of Component

Total neutral sugar contents were determined again using the phenol-sulfuric acid method [76]. Then, 50 μg/mL NHE solution was reacted with 0.5% aqueous phenol solution and concentrated H_2_SO_4_ in a tube for 10 min. The mixture was reacted in boiling water for 15 min, and the absorbance was determined at 490 nm.

Total acid sugar (uronic acid) contents were measured using the m-hydroxyphenyl colorimetric method [77], using galacturonic acid as standard. In an ice bath, 100 μg/mL NHE solution was mixed with 0.2% Na_2_B_4_O_7_/H_2_SO_4_ and oscillated fully. The mixture was reacted in boiling water for 15 min and then added to 1.5 mg/mL m-hydroxyldiphenyl/NaOH after cooling. The absorbance of the oscillated mixture was measured at 523 nm.

Protein contents were determined via Coomassie Brilliant Blue G-250 (GBB) method [78], using bovine serum albumin as the standard. The NHE solution was reacted with 0.1 mg/mL GBB/H_3_PO_4_ for 5 min at 25 °C. The absorbance was measured at 595 nm.

The polyphenol contents of NHE were measured using the Folin–Ciocalteu method [79], using gallic acid as the standard. The NHE solution was reacted with Foline-phenol/Na_2_CO_3_ for 40 min at 40 °C. The absorbance of the mixture was measured at 760 nm.

### 3.5. Characterization of NHE by HPLC and FT–IR

Pre-column derivatization was applied to analyze the monosaccharide composition of NHE [80]. The NHE (2 mg) completed the hydrolysis to monosaccharides under 2 M of trifluoroacetic acid (TFA) for 4 h at 120 °C in a small ampule. The TFA was subsequently dried using the nitrogen-blowing method with methanol, followed by resolution with 2.0 mL of water. The hydrolysate and monosaccharide standard solutions were derivatized by PMP methanol solution at 70 °C for 1 h. After adjusting pH to neutral, 1.0 mL of chloroform was added in sequence, vortexed and mixed for 1 min, the mixtures were centrifuged for 5 min. The lower layer was discarded and the addition was repeated in the upper layer. Finally, the extraction with chloroform was performed twice to obtain the derivative solution in the upper layer.

The derivative solutions were analyzed by the HPLC system (Nexera LC-20AD) using an Agilent C18 column (4.6 mm × 200 mm, 5 μm) after passing through a 0.22 μm microporous membrane. Elution was carried out with a mixture of 0.1 M phosphate buffer (pH 6.7) and acetonitrile (83:17), at a flow rate of 1.0 mL/min. The temperature of the column was set at 30 °C.

The NHE was ground with potassium bromide (KBr) powder of spectroscopic grade in an agate mortar (1:100), milled to allow it to be mixed completely and pressed into a sheet for transmittance. Finally, the organic functional groups of NHE were identified by FT–IR spectrometry using the FT–IR spectrometer (Shimadzu Corporation, Kyoto, Japan) with a scanning wavelength range of 4000~500 cm^−1^ [81].

### 3.6. Thermal (TG–DTG) Analysis of NHE

Thermal analysis was performed to investigate the thermal properties of NHE. About 5 mg of NHE was kept under a nitrogen atmosphere with a heating rate of 20 °C/min from 30 to 800 °C, using a Thermalgravimetric Analyzer (TGA5500, TA Instruments, New Castle, DE, USA).

### 3.7. Antioxidant and Anti-Inflammatory Activity of NHE

According to validated methods [82,83], briefly, 1.0 mL of 0.1 mM DPPH in ethanol was mixed with 1.0 mL of different concentrations of the sample in water. After 30 min incubation in the dark, the DPPH radical scavenging activity was calculated by absorbance at 517 nm using vitamin C (VC, Adamas, Rockville, MD, USA) as the positive control. 

The anti-inflammatory effect of NHE was exerted by inhibiting the activity of hyaluronidase, using dipotassium glycyrrhizinate (Dg, Macklin, Shanghai, China) as the positive control according to Mancarz’s method [84]. Briefly, the hyaluronidase activity of samples was determined using sodium hyaluronate as substrate in an acetic acid buffer containing calcium ions. The absorbance was measured at 585 nm and the inhibitory activity was calculated by 4-Dimethylaminobenzaldehyde.

### 3.8. Cells Culture and Cytotoxicity Assay of NHE

Human Immortalized Epidermal Cells (HaCaT) and mouse melanoma cells (B16) were respectively cultured in DMEM medium (with 1% P/S) supplemented with 10% fetal bovine serum (FBS) with a 5% CO_2_ atmosphere at 37 °C in a humidified incubator. The cytotoxicity of NHE was determined by CCK-8 assays according to the method of instructions [85]. The cell suspension (10^4^ cells/well) was seeded on a 96-well flat-bottomed plate for 24 h in a humidified incubator. Then the medium was replaced with a fresh medium containing different concentrations of NHE (0~2.2 mg/mL) After incubation for 24 h and 48 h, the medium was replaced with a CCK-8 working solution. The cells were incubated in a humidified incubator for 2 h. The optical density (OD) value was measured at 450 nm wavelength by ELISA microplate reader (Thermo Fisher, Waltham, MA, USA), and the percentage of HaCaT or B16 cells activity was calculated.

### 3.9. Measurement of ROS Production

The fluorescent probe DCFH–DA is usually used as a cell-permeative indicator for reactive oxygen species. It can freely cross the cell membrane and enter cells and then be hydrolyzed to generate DCFH, which can be further oxidized to produce the fluorescent DCF in the presence of ROS. Finally, the fluorescent plots are easily detected with a microscope [86].

H_2_O_2_ was used to induce the intracellular production of ROS [87]. The HaCaT cells were stimulated with a nominal concentration of H_2_O_2_ (100 μM) for 2 h. Intracellular accumulation of ROS was detected using detection kit NO S0033S (Beyotime, Shanghai, China). The cells were seeded in black sterile 96-well plates for 24 h to 80% confluence, then cultured with different concentrations of NHE (0.2~1.8 mg/mL) and were stimulated with 100 μM of H_2_O_2_ for 2 h. The basic DMEM medium without other substances was used as the control group. Meanwhile, the medium with 10 μg/mL VC was used as a positive control group. They were incubated together with DCFH–DA fluorescent probe (10 µM) in darkness at 37 °C for 30 min. The fluorescence intensity was measured at the excitation wavelength of 488 nm and the emission wavelength of 525 nm [5]. Parallelly, fluorescence-excited DCF can be observed in plots by a microscope (Axio Vert. A1, Zeiss, Oberkochen, Germany).

### 3.10. Measurement of Melanin Content

According to the method reported by Teng et al. [67] the whitening effect of NHE could be indicated by the melanin content of B16 melanoma cells. B16 melanocytes could rapidly differentiate and produce melanin under the stimulation of α-melanocyte-stimulating hormone (α-MSH), which is an important basis for the construction of the experimental model [88]. Different concentrations of NHE (0~1.0 mg/mL) and kojic acid (20 μg/mL) as positive groups were added to the medium. The B16 cells (10^6^ cells/well) were seeded in sterile 6-well plates with basic medium (no FBS) containing α-MSH (0.25 μg/mL) and cultured for 72 h. After separation with trypsinization, the cells were disrupted by 10% dimethyl sulfoxide (DMSO) with 1 M NaOH at 80 °C for 60 min. The OD value was determined at 405 nm, which indicated the level of melanin in B16 cells using the basic DMEM medium without other substances as the control group.

### 3.11. Wound Healing Scratch Migration Assay

HaCaT cells were trypsinized at 80% confluence and counted with a cell viability analyzer (Vi-CELL XR, Beckman Coulter, CA, USA). A cell suspension was prepared, and the cells were seeded in sterile 6-well plates according to the density of 10^6^ cells/well, then incubated with a 5% CO_2_ atmosphere at 37 °C in a humidified incubator for 24 h. After visualizing under a microscope to establish the formation of monolayer cells in each well, scratches were induced in the monolayers across the diameter of the wells by using a sterile 200 μL pipet-tip [89]. The culture medium with free FBS of the sample group was mixed with different concentrations of NHE (0.2~1.8 mg/mL) and additive-free DMEM medium as the control. For the duration of the experiments, the cells were incubated in a humidified environment with 5% CO_2_ at 37 °C. The photos were taken with a microscope. The wound surface area was measured with ImageJ 1.52i software. According to the following modified equation, the migration rate was calculated and analyzed [73]:

$$\;\mathrm{Migration}\;\mathrm{rate}(\%)=\left[\frac{A_{0}-A_{1}}{A_{0}}\right]\times 100$$

where *A*_0_ is the initial pre-migration wound area (pixels) at time 0 h, and *A*_1_ is the migration wound area (pixels) at 24 h or 48 h.

### 3.12. Statistical Analysis

Design-Expert software 10.0.7 was used in RSM analyses. Other statistical analyses were performed with OriginPro 2021. All the experiments were performed in triplicate. The significance represented that the sample group compared with the control group, *p* < 0.05, *p* < 0.01 and *p* < 0.001 were performed *, ** and ***, respectively. The data were presented as mean (*n* ≥ 3) ± standard deviation (error bars). The data on the cell experiment were presented as mean (*n* ≥ 3) ± standard deviation (error bars).

## 4. Conclusions

The ultrasound-assisted cellulase extraction method was employed to optimize the extraction process of *Nymphaea hybrid* flowers for bioactive water extraction. Under the optimized conditions using the RSM model, including an ultrasound time of 30.69 min, a liquid-to-solid ratio of 32.69 mL/g, enzymatic hydrolysis temperature of 47.15 °C and enzyme content of 3%, the actual yield of total sugar was 8.11 ± 0.08%. This is the first time that the bioactive water extracts have been systematically obtained from *Nymphaea hybrid*, comparatively, the product obtained has better applicability and lower production cost. The 10 monosaccharide compositions of NHE were identified and quantified by HPLC with the PMP derivation method. Thermal properties and stability were measured using the TG–DTG method and the result indicated that NHE had a relative thermal stability below 275.0 °C and a wide range of applications. The chemical evaluation assays and the intracellular ROS simulation assays in HaCaT cells verified that NHE behaved with the potential as an antioxidant to scavenge free radicals and protect the cells against H_2_O_2_-induced oxidative damage. Moreover, the inhibiting hyaluronidase assays via the Elson-Morgan method and scratch assays on HaCaT cells proved that the NHE behaved with anti-inflammatory and wound repair ability. The whitening effect assessed on B16 cells proved that NHE can inhibit melanin production. Overall, NHE could be developed as an active ingredient with comprehensive functionality including whitening, anti-inflammatory, protective effects against oxidative damage and the ability to promote wound healing, which promotes a prospect as a functional raw material in the field of cosmetics and food.

## Figures and Tables

**Table 1:** Box–Behnken experimental design and results.

Run	A	B	C	Yield (%)
1	40	20	50	6.47 ± 0.03
2	30	30	50	8.11 ± 0.08
3	20	40	50	6.55 ± 0.11
4	40	40	50	7.31 ± 0.09
5	30	20	55	6.79 ± 0.09
6	40	30	45	7.22 ± 0.13
7	30	20	45	7.14 ± 0.18
8	30	30	50	7.98 ± 0.08
9	30	30	50	8.16 ± 0.09
10	30	30	50	8.20 ± 0.21
11	30	40	45	7.75 ± 0.15
12	30	40	55	6.63 ± 0.09
13	30	30	50	7.88 ± 0.12
14	40	30	55	6.74 ± 0.13
15	20	30	45	7.35 ± 0.05
16	20	20	50	6.19 ± 0.14
17	20	30	55	6.04 ± 0.09

**Table 2:** Variance analysis of regression model results.

Variables	Sum of Squares	df	Mean Square	F-Value	*p*-Value Prob. > F
Model	8.01	9	0.89	34.47	<0.0001 ***
A	0.33	1	0.33	12.73	0.0091 **
B	0.34	1	0.34	13.16	0.0084 **
C	1.33	1	1.33	51.54	0.0002 ***
AB	0.058	1	0.06	2.26	0.0176 *
AC	0.17	1	0.17	6.70	0.0360 *
BC	0.15	1	0.15	5.64	0.0492 *
A^2^	2.95	1	2.95	114.36	<0.0001 ***
B^2^	1.51	1	1.51	58.55	0.0001 ***
C^2^	0.64	1	0.64	24.78	0.0016 **
Residual	0.18	7	0.035	2.00	
Lack of fit	0.11	3	0.036	34.47	0.2558
Pure error	0.072	4	0.034		

R^2^ = Coefficients of determination. Significance: * *p* < 0.05, ** *p* < 0.01 and *** *p* < 0.001.

**Table 3:** The component and content of NHE.

Physicochemical Property	Proportion (*w*/*w*%)
Neutral sugar	41.31 ± 0.24
Uronic acid	41.68 ± 0.42
Protein	0.09 ± 0.01
Polyphenol	2.20 ± 0.11
Yield	8.22 ± 0.16

**Table 4:** The monosaccharide composition of NHE.

Monosaccharide Composition	Peak Area (%)
Man	4.555
Rib	0.139
Rha	5.655
GlcUA	3.946
GalUA	47.311
Glc	6.811
Gal	18.612
Xyl	6.323
Ara	6.382
Fuc	0.265

## Data Availability

The data that support the findings of this study are available from the corresponding author, upon reasonable request. Samples of the extraction of *Nymphaea hybrid* are available from the authors.
